# Imaging Immune and Metabolic Cells of Visceral Adipose Tissues with Multimodal Nonlinear Optical Microscopy

**DOI:** 10.1371/journal.pone.0038418

**Published:** 2012-06-11

**Authors:** Yasuyo Urasaki, Mary G. Johlfs, Ronald R. Fiscus, Thuc T. Le

**Affiliations:** 1 Human Health and Environment Program, Desert Research Institute, Las Vegas, Nevada, United States of America; 2 Department of Drug Development, Nevada Cancer Institute, Las Vegas, Nevada, United States of America; 3 Center for Diabetes and Obesity Prevention, Treatment, Research and Education, and the College of Pharmacy, Roseman University of Health Sciences, Henderson, Nevada, United States of America; The University of Queensland, Australia

## Abstract

Visceral adipose tissue (VAT) inflammation is recognized as a mechanism by which obesity is associated with metabolic diseases. The communication between adipose tissue macrophages (ATMs) and adipocytes is important to understanding the interaction between immunity and energy metabolism and its roles in obesity-induced diseases. Yet visualizing adipocytes and macrophages in complex tissues is challenging to standard imaging methods. Here, we describe the use of a multimodal nonlinear optical (NLO) microscope to characterize the composition of VATs of lean and obese mice including adipocytes, macrophages, and collagen fibrils in a label-free manner. We show that lipid metabolism processes such as lipid droplet formation, lipid droplet microvesiculation, and free fatty acids trafficking can be dynamically monitored in macrophages and adipocytes. With its versatility, NLO microscopy should be a powerful imaging tool to complement molecular characterization of the immunity-metabolism interface.

## Introduction

Obesity is an established risk factor for an array of medical problems including insulin resistance, type 2 diabetes, fatty liver diseases, cardiovascular diseases, neurodegenerative disorders, and some cancers [1]–[3]. Although the mechanism linking obesity to related diseases is not clearly understood, increasing evidence from the literature points to the inflammation of visceral adipose tissues (VATs) as a potential cause [4]. Central to adipose tissue inflammation in obesity is the increased accumulation of adipose tissue macrophages (ATMs) [5], which exhibit morphological polarization parallel with the progression of obesity [6]–[7]. In lean adipose tissues, ATMs are mostly in the M2 state and express anti-inflammatory markers such as arginase and interleukin 10 [6]. On the contrary, in obese adipose tissues, ATMs are mostly in the M1 state and express pro-inflammatory markers such as tumor necrosis factor α (TNF α), interleukin 6 (IL-6), and inducible nitric oxide synthase (iNOS) [6], [8]. It has been proposed that the paracrine loop between ATMs and adipocytes regulates the phenotypic switch of ATMs [6]. However, it remains unclear how this ATMs phenotypic switch affects the function of ATMs or the adipocyte-macrophage communication during obesity development.

A significant challenge to the studies of adipocyte-macrophage communication is the ability to dynamically monitor the phenotype and function of adipocytes and ATMs in complex tissues. In this paper, we employ a multimodal NLO microscope to characterize the composition of VATs, including adipocytes, ATMs, and collagen fibrils, of lean and obese mice. We have previously described the versatility of this NLO microscope for complex tissues visualization and analysis with multiple imaging modalities including coherent anti-Stokes Raman scattering (CARS), second harmonic generation (SHG), and two-photon fluorescence (TPF) [9]–[10]. Here, we demonstrate the potential of this multimodal NLO microscope to examine metabolic and immune cells of VATs.

## Results

**Figure 1:**
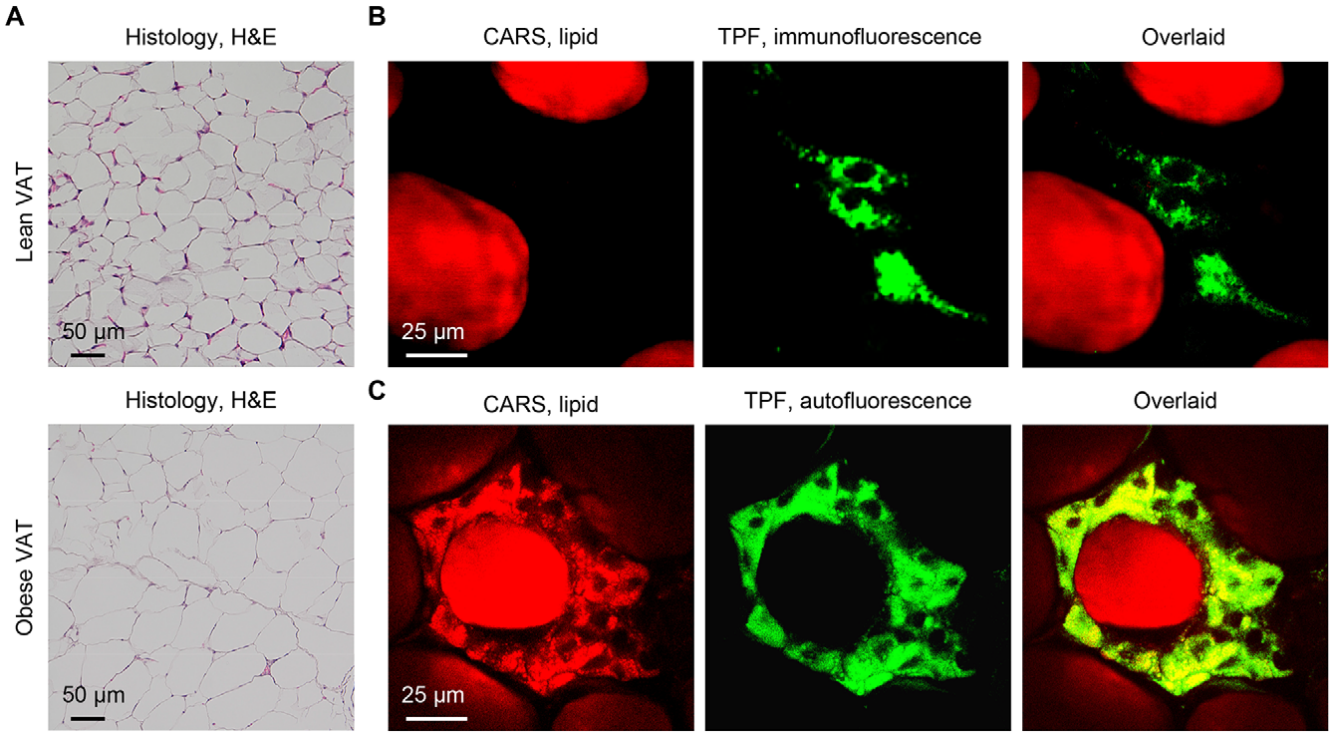
Imaging the visceral adipose tissues (VATs) of lean and obese mice with NLO microscopy. (A) Histology of lean (upper panel) and obese (lower panel) VATs. (B) CARS imaging of lipid droplets of adipocytes (red) and two-photon fluorescence (TPF) imaging of preadipocytes (green) immunolabeled with FITC-conjugated antibodies to Pref-1 of a lean VAT. (C) CARS imaging of lipid droplets (red) and two-photon autofluorescence imaging of unidentified cells (green) surrounding an adipocyte of an obese VAT.

We first examined the composition of VATs in lean and obese mice with histology and CARS imaging (**Figure 1**). From the histology images, the diameters of the lipid droplets of adipocytes in the VATs of lean mice of ∼53 µm were approximately one-half of those in the VATs of obese mice of ∼98 µm (**Figure 1A**). Using CARS microscopy to examine the explanted VATs of lean mice, we found that CARS signal of lipid arose mainly from the lipid droplets of adipocytes. Other cell types, such as preadipocytes, can only be identified with immunolabeling with antibodies to preadipocyte marker Pref-1 [11] and two-photon fluorescence (TPF) imaging (**Figure 1B**, **upper panels**). In contrast, in explanted VATs of obese mice, we found that CARS signal of lipid arose from both the lipid droplets of adipocytes and the lipid droplets of other cells surrounding adipocytes. Interestingly, the lipid-rich cells surrounding adipocytes also exhibited strong intrinsic autofluorescence with TPF imaging using a 510/42 nm emission filter (**Figure 1B**, **lower panels**). Indeed, strong autofluorescence was observed for all non-adipocyte cells that were lipid-rich in the VATs of obese mice. Such autofluorescence was not observed in any non-adipocyte cell that was lipid-poor in the VATs of lean or obese mice (data not shown). Generally, adipocytes of both lean and obese mice do not exhibit any autofluorescence in the 490 nm to 530 nm range (**Figure 1B**).

**Figure 2:**
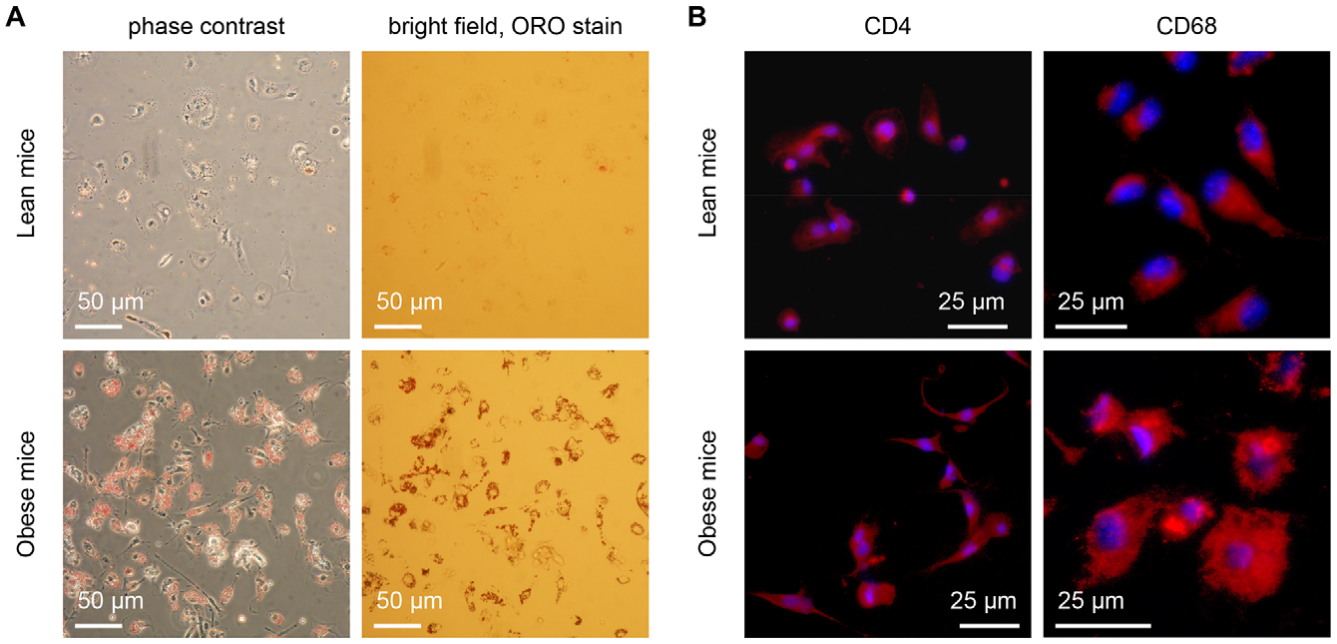
Lipid-rich adipose tissue macrophages (ATM) identified with Oil Red O (ORO) staining and immunofluorescence imaging. (A) Phase contrast and bright field images of isolated VAT stromal cells stained with ORO. (B) Immunofluorescence images of isolated VAT stromal cells stained with conjugated antibodies to CD4 and CD68 cell surface markers for macrophages.

To determine the identity of the lipid-rich autofluorescence cells of the VATs, we isolated the stromal cells of the VATs for further analysis. Using oil red O (ORO) to stain for lipid droplets, we found that less than one percent of the stromal cells isolated from the VATs of lean mice exhibited ORO staining (**Figure 2A** **, upper panels**). In contrast, more than eighty percent of the stromal cells isolated from the VATs of obese mice exhibited strong ORO staining (**Figure 2A** **, lower panels**). This observation indicated a significant increase in the intracellular lipid droplet accumulation in non-adipocyte cells of the VATs of obese mice as compared to the VATs of lean mice. Interestingly, more than eighty percent of the stromal cells from the VATs of both lean and obese mice positively stained for CD4 and CD68 surface markers (**Figure 2B**) [12]–[13]. Thus, lipid-rich autofluorescent cells in the VATs of obese mice were likely lipid-rich ATMs.

**Figure 3:**
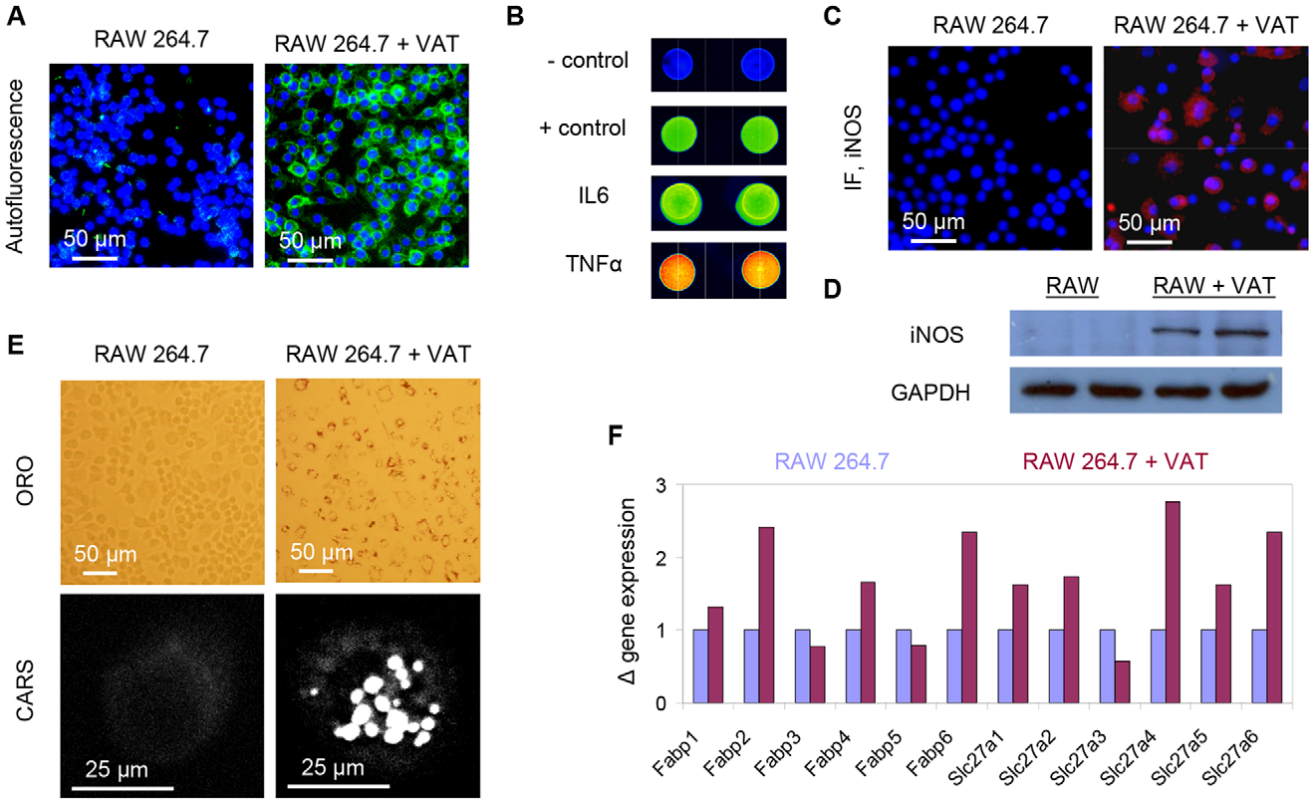
Potential source of autofluorescence and lipid in macrophages. (A) Widefield fluorescence images of RAW 264.7 macrophages (left panel) and RAW264.7 macrophages in co-culture with explanted VATs (right panel). (B) Peptide array to identify adipose tissue secreted pro-inflammatory cytokines. (C) Immunofluorescence (IF) imaging and (D) Western blot analysis revealed increased expression of iNOS in RAW264.7 macrophages co-cultured with explanted VATs as compared to control RAW264.7 macrophages. (E) RAW264.7 macrophages co-cultured with explanted VATs accumulated intracellular lipid droplets observable with ORO staining (upper right panel) and CARS imaging (lower right panel). (F) Real time PCR analysis of gene expression of fatty acid binding proteins (encoded by Fabp1-6 genes) and fatty acid transport proteins (encoded by Slc27a1-6 genes) in control RAW264.7 macrophages and RAW264.7 macrophages co-cultured with VATs.

To investigate the source of autofluorescence and lipid in ATMs, we studied a co-culture of RAW264.7 macrophages and explanted VATs. We found that incubation with explanted VATs induced strong autofluorescence in RAW246.7 cells (**Figure 3A**). Cytokine array analysis of the VATs' conditioned media revealed a strong presence of pro-inflammatory cytokines such as IL6 and TNFα (**Figure 3B**). In addition, both immuno-staining and Western blot analysis showed that RAW264.7 macrophages highly expressed iNOS when co-cultured with VATs (**Figure 3C, D**). Hence, VATs appeared to induce both autofluorescence and activation of RAW246.7 macrophages. The enzyme iNOS requires 5 co-factors for its catalytic activity including flavin adenine dinucleotide (FAD), flavin mononucleotide (FMN), heme, tetrahydrobiopterin, and calmodulin [14]. Both FAD and FMN exhibit fluorescence that peaks around 520 nm which overlapped with our detection wavelengths [**15**]. It is plausible that elevated iNOS expression level in activated RAW264.7 macrophages contributed to the observed autofluorescence.

Using both ORO staining and label-free CARS imaging of RAW264.7 macrophages, we found that incubation with VATs also induced intracellular lipid droplet accumulation (**Figure 3E**). Real-time PCR gene expression profiling revealed that incubation with VATs also induces increased expression of selective isoforms of fatty acid binding proteins (FABPs) and fatty acid transport proteins (FATPs) in RAW264.7 macrophages (**Figure 3F**). FABPs and FATPs facilitate the uptake and transport of free fatty acids into cells [16]–[17]. It is plausible that these proteins contributed to the uptake of free fatty acids from the cultured medium into RAW264.7 macrophages. Taken together, our co-culture experiments provided evidence that supports lipid-rich autofluorescent cells in VATs as lipid-rich ATMs.

**Figure 4:**
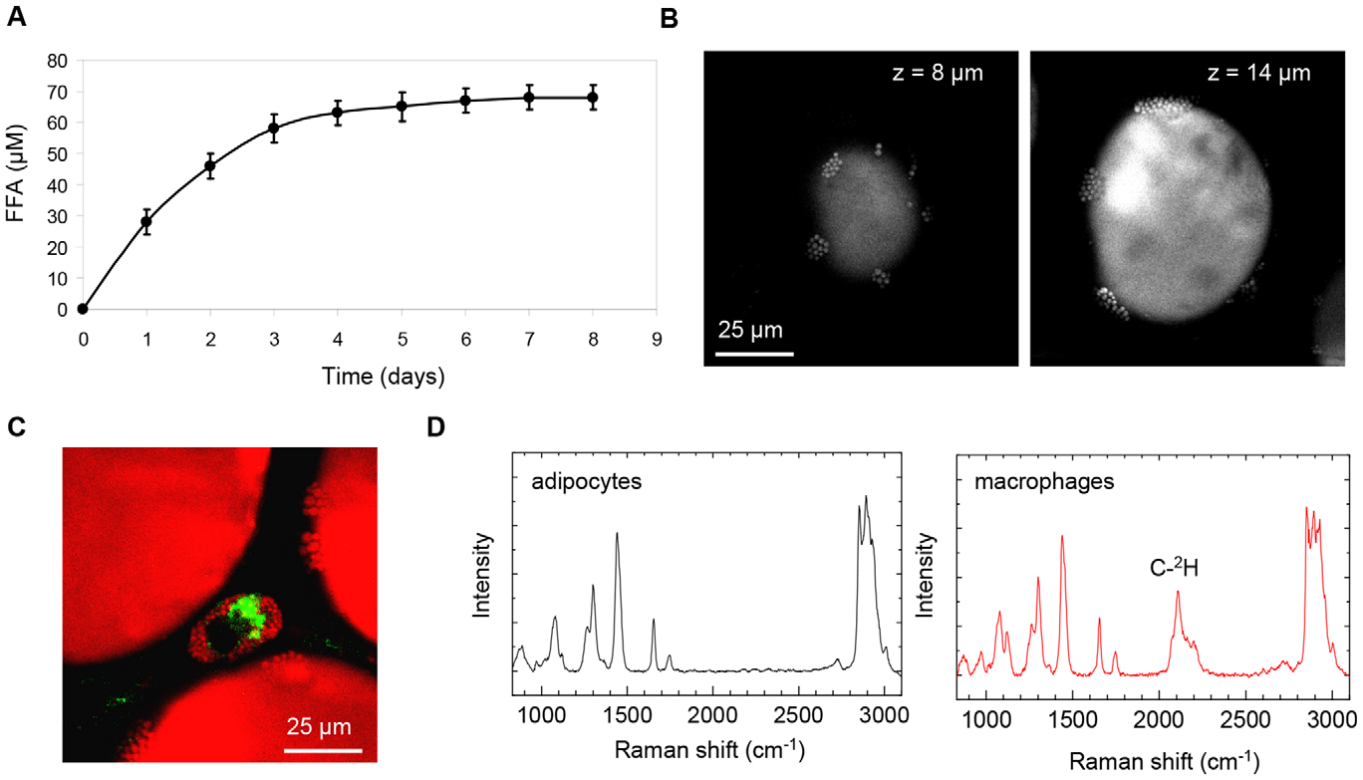
Evidence for lipid mobilization from adipocyte and uptake of exogenous lipid by ATMs. (A) Free fatty acids (FFA) concentration of the VAT conditioned medium as a function of incubation time. Error bars represent standard deviation across triplicate measurements. (B) Evidence of lipid droplet microvesiculation revealed by CARS imaging. (C) An autofluorescent and lipid-rich ATM was observed in proximity of adipocytes with lipid droplet microvesiculation. (D) Monitoring the trafficking of exogenous deuterated palmitic acid with spontaneous Raman microspectroscopy. Absence and presence of C-^2^H peak in the lipid droplets of adipocytes and ATMs, respectively, were observed.

VATs serve as major energy depots in the body. When maintained *ex vivo*, VATs secreted up to 70 µM of free fatty acids into the cultured medium (**Figure 4A**). To visualize how adipocytes mobilized energy, we closely examined the morphology of the lipid droplets. After 24 hours in cultured media, we observed the formation of many small patches of micron-size lipid droplets on the surface of large monocular lipid droplets of adipocytes (**Figure 4B**). Lipid droplet microvesiculation could be a consequence of lipid droplet fragmentation [18]–[19] or re-esterification of free fatty acids [20]–[21]. Interestingly, lipid droplet microvesiculation in adipocyte was observed together with lipid-rich autofluorescent ATMs (**Figure 4C**). It is plausible that ATMs imported free fatty acid secreted into the tissue microenvironment by adipocytes.

To monitor the trafficking of free fatty acids, we added 50 µM deuterated d-31 palmitic acid into the cultured media. Deuterated lipid can be clearly distinguished from natural lipid due to the presence of a strong carbon-deuterium (C-^2^H) Raman signature at ∼2150 cm^−1^ [22]. Using spontaneous Raman microspectroscopy, we detected the presence of deuterated lipid in the lipid droplets of ATMs (**Figure 4D**) at 24 hours after the addition of d31-palmitic acid. This observation strongly indicated that ATMs were importers of exogenous free fatty acids. On the other hand, no deuterated lipid was detected in the lipid droplets of adipocytes (**Figure 4D**). It is plausible that the concentration of imported d31-palmitic acid in adipocytes was miniscule compared to the concentration of endogenous lipid. Hence, imported d31-palmitic acid was below the detection limit of spontaneous Raman microspectroscopy.

**Figure 5:**
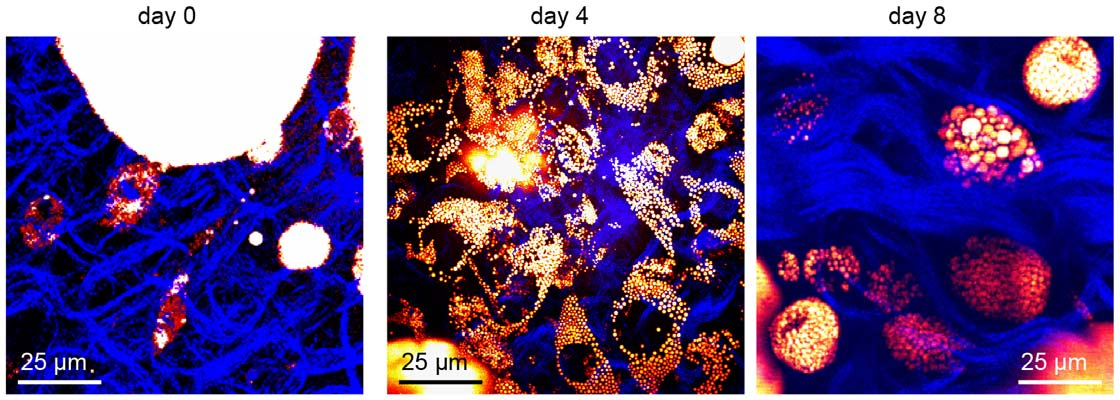
Imaging the kinetics of lipid accumulation in ATMs of explanted VATs. Steady accumulation of lipid droplets in ATMs was observed with CARS microscopy as a function of time in explanted VATs. Collagen fibrils visualized with second harmonic generation were displayed in blue.

We further examined the morphology of ATMs as a function of time in explanted VATs. We observed that ATMs accumulated intracellular lipid droplets over time with steady increase in both lipid droplet number and size (**Figure 5**). The cell shape of ATMs also changed from spindle to round, which could be a consequence of excess free fatty acids in the microenvironment incorporating onto the cellular membrane [23]. Given the scavenging role of ATMs, the presence of lipid-rich ATMs could be a strong indicator of excess lipid in the VAT microenvironment.

**Figure 6:**
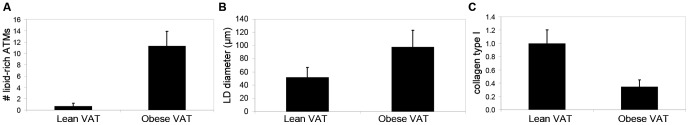
Quantitative analysis of VAT composition in lean and obese mice. (A) Average number of lipid-rich ATMs per analysis volume with xyz dimensions of 250 µm×250 µm×50 µm. (B) Average diameter of lipid droplets of ∼200 adipocytes for lean VATs and ∼100 adipocytes for obese VATs. (C) Normalized level of collagen fibrils type I, as measured by SHG intensity, in the VATs of lean and obese mice. Data was normalized to 1 for lean VATs and respectively for obese VATs. Error bars represents standard deviation across 81 volumes analyzed.

Finally, we applied multimodal NLO microscopy to systematically analyze the composition of VATs of lean and obese mice. Consistent with the observations described in Figure 1 and 2, we found a more than 10-fold increase in the number of lipid-rich ATMs in the VATs of obese mice versus lean mice (**Figure 6A, B**). Consistent with the histology data and the literature [5], the diameters of the lipid droplets of adipocytes in the VATs of lean mice were approximately one-half of those in the VATs of obese mice (**Figure 6C**). Using second harmonic generation to quantify collagen fibril type I of the VATs [24]–[25], we found a 3-fold reduction in obese mice versus lean mice (**Figure 6D**). Taken together, our data revealed a significant change in the composition of VATs in obese mice versus lean mice. Most importantly, we showed that such compositional analysis of VATs can be achieved in a label-free manner with multimodal NLO microscopy.

## Discussion

The regulation of metabolic and immune response is highly conserved and coordinated [26]. Nuclear receptors such as the peroxisome proliferator activated receptors and liver X receptors which are activated by free fatty acids and cholesterol metabolites, exert regulatory control over the expression of a wide range of both metabolic and inflammatory genes [27]. Both adipocytes and macrophages have large capacity to store intracellular lipids, have extensive overlapping in the expression of metabolic and inflammatory genes, and secrete similar cytokines [4], [26]. Furthermore, metabolic cells such as adipocytes or hepatocytes are always within proximity of the immune cells such as macrophages, Kuppfer cells, and lymphocytes. The conserved organization and regulation of metabolic and immune cells support their functional dependence and help to explain the correlation between metabolic dysfunction of adipocytes and impaired inflammatory response of macrophages in obesity [4], [26].

Critical for the functional integration of the metabolic and immune systems is the dynamic communication between adipocytes and ATMs. Yet adipocyte-ATM communication is among one of the least studied systems. In this paper, we demonstrated the capability of a multimodal NLO microscope system to examine the composition of explanted VATs. We showed that CARS imaging allowed visualization of lipid droplets within adipocytes and ATMs. Lipid-rich ATMs also had strong autofluorescence which could be visualized with TPF imaging. Combined CARS, TPF, and SHG imaging permitted label-free imaging of adipocytes, ATMs, and collagen fibrils type I of explanted VATs, respectively. In addition, integrated spontaneous Raman microspectroscopy also allowed monitoring of deuterated lipid trafficking into ATMs. Together, we showed that multimodal NLO microscopy could become a powerful label-free visualization means to study adipocytes and ATMs of the explanted VATs. Multiphoton microscopy has been widely applied for intravital imaging of small animals [28]. It is conceivable that the studies of adipocyte-macrophage communication can also be examined *in vivo* through invasive surgery or implantation of adipose tissues in intravital imaging chambers [29]–[30].

We observed possible integration between immunity and metabolic activity in macrophages. For example, activated RAW264.7 macrophages exhibited increased iNOS expression together with intracellular lipid droplet accumulation. It remains unclear how the normal functions of macrophages such as phagocytosis and reverse cholesterol transport are affected by activated macrophages. However, lipid-rich macrophages are frequently detected in the obese VATs or atherosclerotic lesions and have been hypothesized to play a critical role in the etiology of metabolic diseases. It appears that increased iNOS expression in macrophages was observed together with increased autofluorescence around 520 nm. Previously, we have also reported autofluorescence of atherosclerotic foam cells around 520 nm and attributed the source to oxidized low-density lipoprotein [31]. A number of other co-factors including NADH and NADPH within cells also exhibit autofluorescence that overlap with this wavelength [32]. While it is difficult to accurately identify a single source for the observed autofluorescence, it is clear that autofluorescence around 520 nm can be used as a detection marker for activated macrophages. The intrinsic autofluorescence of lipid-rich ATMs can interfere with immunofluorescence in the green spectrum (490 nm–530 nm). Therefore, we recommend the use of antibodies conjugated with red or far-red fluorescent dyes (>570 nm) for immunolabeling of ATMs.

Increased iNOS expression in cells has been shown to affect the NO/cGMP/PKG signaling pathway which subsequently affects cell proliferation and metabolic activity [33]–[34]. Particularly, increased intracellular nitric oxide concentration has been shown to promote intracellular lipid droplet accumulation in metabolic cells [35]–[36]. Further investigation of the role of nitric oxide in macrophages could shed light on the correlation between inflammation and metabolic activity, as indicated by increased iNOS expression and autofluorescence and the accumulation of intracellular lipid droplets, respectively. The ability of multimodal NLO microscopy to dynamically monitor lipid-rich macrophages in tissue microenvironment should be a powerful complement to biochemical assays to study the function, metabolic activity, and inflammatory activation of macrophages in healthy and diseased tissues.

## Materials and Methods

### Multimodal NLO microscopy

The signal (924.2 nm) and idler (1255 nm) outputs of an optical parametric oscillator (OPO, Levante Emerald, Berlin, Germany) were used as the pump and Stokes beams, respectively, to produce a frequency difference of 2851 cm^−1^. The OPO was synchronously pumped by the second harmonic output (532 nm) of a mode-locked Nd: YVO4 laser (HighQ Laser, Austria). The pump and Stokes beams were inherently synchronized and collinearly overlapped at the exit port of the OPO. The laser beams were passed through a laser scanner (C1plus, Nikon, Melville, NY) and focused with a 60× IR objective into the sample. The combined laser power was attenuated with neutral density filters to 66 mW at sample. The same excitation laser source was used for simultaneous CARS and TPF imaging. Epi-reflected signal was directed into a multi-channel detector, spectrally separated with dichroic mirrors, selected with bandpass filters (Semrock, Lake Forest, IL), and detected with red-sensitive photomultiplier tubes (R10699, Hamamatsu, Japan). Bandpass filters for SHG signal, autofluorescence signal, and CARS signal were 465/30 nm, 510/42 nm, and 736/128 nm, respectively.

### NLO image acquisition and analysis

Images were acquired at approximately 1 second per frame. For VATs composition analysis, 51 frames taken along the vertical axis at 1-micron intervals were acquired to obtain 3D volumes of 250 µm×250 µm×50 µm in xyz dimensions. Generally, 9 volumes were obtained per VAT tissue examined. For display purpose, stacked 3D images were presented. For quantitative analysis of collagen fibrils using SHG signal, total integrated SHG intensity for 31 frames was obtained for each volume [25]. Total integrated SHG intensity of VATs for 9 mice with 9 volumes analyzed per mouse (or 81 volumes total) was used to compare the collagen fibril content of lean versus obese VATs. For quantitative analysis of lipid-rich ATMs, visual inspection and enumeration of lipid-rich macrophages was performed in 81 volumes of each animal group. The diameters of adipocytes were measured using the particle sizing function provided by the ImageJ software on 3-D stacked images as described previously [37].

### Raman microspectroscopy

A continuous-wave laser at 671 nm (Laserglow, Toronto, Ontario, Canada) was collinearly combined with CARS laser beams using a combiner mirror (Chroma, Bellows Falls, VT) and served as the excitation light source for Raman microspectroscopy. A spectrometer (Shamrock SR-303i-A, Andor Technology, Belfast, U.K.) was mounted to the backport of the microscope (Ti-E, Nikon, Melville, NY). Confocal Raman was achieved by selecting scattered light with a bandpass filter and focusing it with a 100-mm focal length achromatic lens into a 50-micron pinhole. The spectrometer is equipped with a 300 grooves/mm 500-nm blaze angle grating and a thermoelectrically cooled back-illuminated electron-multiplying charge-coupled device (EMCCD; Newton DU970N-BV, Andor Technology, Belfast, U.K.). The laser power at sample was attenuated to 20 mW with neutral density filters. Raman data were collected by averaging three 4-second acquisitions.

### Cell culture microscopy

Bright field, phase contrast, and wide field fluorescence imaging was performed on a CKX41 inverted microscope (Olympus, Center Valley, PA). Illumination and fluorescence light source was provided by a 30 W halogen lamp and a 50 W Mercury lamp, respectively. Fluorescence cubes ultraviolet, blue, and green were used for DAPI, FITC or autofluorescence, and tetramethylrhodamine visualization respectively.

### Animals

A group of 24 male C57BL mice at 4–6 weeks old was purchased from the Jackson Laboratory (Bar Harbor, Maine). Half of the mice were fed with a high fat diet (Cat. No. TD.88137, Harlan Laboratories, Indianapolis, IN) with 42% fat and 4.5 Kcal/g. The other half were fed with a lean diet (Cat. No. 7001, Harlan Laboratories, Indianapolis, IN) with 4.25% fat and 3 Kcal/g for 16 weeks. Visceral adipose tissues (VATs) were terminally collected for analysis. All animal experiments were done with the approval of the Desert Research Institute Animal Care and Use Committee.

### Maintenance of explanted VATs

VATs were kept in glass-bottom tissue culture dishes (Cat. No. P50G-1.5-30-F, Mattek, Ashland, MA) containing 2 ml of high glucose (4.5 g/L) DMEM media supplemented with 10% fetal bovine serum and antibiotics in an incubator at 37°C and 5% CO_2_. When used for imaging, the dishes were removed from the incubator, placed on the microscope stage at room temperature for 2 hours, and then returned to incubator.

### Isolation of ATMs from VATs

VATs were incubated with 1 mg/ml collagenase (Cat. No. C2139, Sigma-Aldrich, St. Louis, MO) in DMEM supplemented with 10% FBS for 1 hour at 5% CO_2_ and 37°C with frequently stirring. Treated VATs were centrifuged at 125×g for 5 minutes. The stromal cells including macrophages were pelleted out at the bottom. The stromal cells were incubated in DMEM media supplemented with 10% FBS and antibiotics on glass-bottom dishes for 24 hours at 5% CO_2_ and 37°C prior to analysis.

### Antibodies used for immuno-staining

Primary antibodies were rabbit polyclonal antibody to mouse CD4 (Cat. No. ABIN460000, antibodies-online.com, Atlanta, GA) and rat monoclonal antibody to mouse CD68 (Cat. No. HM1070, Hycult Biotech, Plymouth Meeting, PA). Secondary antibodies were purchased from Molecular Probes, Eugene, OR including tetramethylrhodamine goat anti-rabbit IgG (H+L) (Cat. No. T-2769) and tetramethylrhodamine goat anti-mouse IgG (H+L) (Cat. No. T-2762).

### Antibodies used for Western blots

Primary antibodies were rabbit polyclonal antibody to mouse iNOS (Cat. No. ab15323, Abcam Boston, MA) and mouse monoclonal antibody to GAPDH (Cat. No. MAB374, Millipore Billerica, MA). Secondary antibodies were purchased from Pierce Biotechonology, Rockford, IL including ImmunoPure goat anti-mouse (Cat. No. 31430) and/or rabbit IgG (H+L) peroxidase conjugated (Cat. No. 31460).

### Western blots

Total cell extracts were analyzed on 10% SDS-polyacrylamide gel and transferred onto nitrocellulose membrane (Cat. No. BA83, Whatman, Piscataway, USA). Transferred membrane was blocked with 5% milk and 0.1% Tween-TBS. Blocked membrane was incubated with primary antibodies at 4°C for overnight, washed thoroughly, and then incubated with secondary antibodies. Membrane was developed with Enhanced Chemiluminescence (ECL) Reagents (Cat. No. 34075, Thermo Scientific, Rockford, IL).

### Immunostaining

For staining of cells, cells were washed with PBS buffer, and fixed with ice cold methanol for 5 minutes or 4% paraformaldehyde in PBS for 10 minutes and washed with PBS buffer. After permeabilization in 0.2% TritonX in PBS for 5 minutes, cells were blocked with 10% goat serum (Cat. No. PCN5000, Invitrogen, Carlsbad, CA) in 1% BSA/PBS for 1 hour at room temperature. Cells were incubated with primary antibodies in 1% BSA in PBS for overnight at 4°C, then secondary antibodies were applied and mounted with Prolong Antifade Gold reagent with DAPI (Cat. No. P-36931, Invitrogen). For staining of VATs, whole VATs were incubated with 10 µg/ml of isolectin IB4 conjugated with FITC (Cat. No. L2895, Sigma-Aldrich) diluted in minimal DMEM media for 4 hours. Stained tissues were washed thoroughly with minimal DMEM media to remove unstained excess dyes and imaged with CARS microscopy.

### Oil Red O staining

Cells were washed with PBS buffer and fixed with 10% formalin solution for 30 minutes at room temperature. Formalin solution was removed and cells were washed with PBS buffer. Next, cells were incubated with 60% isopropanol for 5 minutes at room temperature. After the removal of isopropanol, Oil Red O (Cat. No. O0625, Sigma-Aldrich) was added for 5 minutes at room temperature. Finally, cells were rinsed with distilled water until the rinsed off water was clear and imaged with bright field microscopy.

*Co-cultures of VATs and RAW264.7 macrophages*. RAW264.7 cells (Cat. No. CL-173, ATCC, Manassas, VA) were grown in 2 ml of RPMI1640 media supplemented with 10% FBS to a density of 0.5 million cells per 35-mm culture dish. Then, freshly collected VATs of obese mice were added to the culture dishes (approximately 1.5 grams of VAT per dish). Co-cultures were maintained in an incubator at 37°C and 5% CO_2_ for 2 days prior to analysis.

### Detection of VAT secreted cytokines

Explanted VATs collected from obese mice were maintained *ex vivo* in 2 ml of DMEM media per 1.5 grams of VAT for 2 days. Conditioned media was collected and assayed for secreted cytokines using an obesity biomarker array according to manufacturer's protocol (Cat. No. OC-KF-003, Phoenix Pharmaceutical, Burlingame, CA).

### Quantitative real time PCR array

Total RNA extracts were reverse transcribed using the RT^2^ PCR array first strand kit (Cat. No. 330401, SABioscieces, Frederick, MD). Mouse fatty acid metabolism PCR array (Cat. No. PAMM007C, SABiosciences) was performed using the RT^2^ SYBR Green qPCR mastermix (Cat. No. 330522, SABiosciences) following manufacturer's instruction on a 7500 Fast Real-Time PCR System (Applied Biosystems, Foster City, CA).

### Free fatty acid quantification

Conditioned media from explanted VATs of obese mice were used for free fatty acid measurement using a quantification kit according manufacturer's protocol (Cat. No. K612-100, Biovision, Milpitas, CA).
